# Infiltrating treg reprogramming in the tumor immune microenvironment and its optimization for immunotherapy

**DOI:** 10.1186/s40364-024-00630-9

**Published:** 2024-09-04

**Authors:** Zhaokai Zhou, Jiaxin Xu, Shutong Liu, Yingying Lv, Ruiqi Zhang, Xing Zhou, Yuyuan Zhang, Siyuan Weng, Hui Xu, Yuhao Ba, Anning Zuo, Xinwei Han, Zaoqu Liu

**Affiliations:** 1 Department of Interventional Radiology, The First Affiliated Hospital of Zhengzhou University, Zhengzhou, Henan 450052 China; 2 Department of Urology, The First Affiliated Hospital of Zhengzhou University, Henan, 450052 China; 3 Department of Human Anatomy, School of Medical Sciences, Zhengzhou University, Zhengzhou, Henan, 450001 China; 4 Department of Pediatrics, The First Affiliated Hospital of Zhengzhou University, Zhengzhou, Henan 450052 China; 5 Department of Pediatric Surgery, The First Affiliated Hospital of Zhengzhou University, Zhengzhou, Henan 450052 China; 6 Interventional Institute of Zhengzhou University, Zhengzhou, Henan 450052 China; 7 Interventional Treatment and Clinical Research Center of Henan Province, Zhengzhou, Henan 450052 China; 8 Institute of Basic Medical Sciences, Chinese Academy of Medical Sciences and Peking Union Medical College, Beijing, 100730 China

**Keywords:** Tumor immune microenvironment, Tumor-infiltrating tregs, Reprogramming, Immunotherapy

## Abstract

Immunotherapy has shown promising anti-tumor effects across various tumors, yet it encounters challenges from the inhibitory tumor immune microenvironment (TIME). Infiltrating regulatory T cells (Tregs) are important contributors to immunosuppressive TIME, limiting tumor immunosurveillance and blocking effective anti-tumor immune responses. Although depletion or inhibition of systemic Tregs enhances the anti-tumor immunity, autoimmune sequelae have diminished expectations for the approach. Herein, we summarize emerging strategies, specifically targeting tumor-infiltrating (TI)-Tregs, that elevate the capacity of organisms to resist tumors by reprogramming their phenotype. The regulatory mechanisms of Treg reprogramming are also discussed as well as how this knowledge could be utilized to develop novel and effective cancer immunotherapies.

## Introduction

**Fig. 1:**
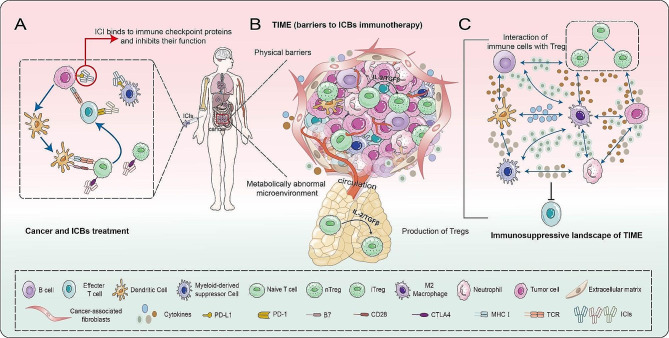
**Immune checkpoint blockades (ICBs) and Tumor immunosuppressive microenvironment.** **A**. ICBs are extensively utilized in the management of a wide range of cancer types. **B**. Tumor immune microenvironment including various immune cells, tumor-associated inflammatory factors, immunosuppressive molecules and extracellular matrix, often causes patients with ‘cold’ tumors, thereby developing resistance and relapse to ICBs. **C**. Cellular crosstalk between Treg and immune cells. ICBs, Immune checkpoint blockades

In recent years, the application of immunology in oncology treatment has risen, mainly facilitating the immune responses against tumors by targeting the immune cell regulatory pathway in tumor immune microenvironment (TIME). For instance, immune checkpoint blockades (ICBs) have been demonstrated to be effective in a fraction of patients, but only a minority achieve long-term clinical efficacy (Fig. 1A). Individuals with ‘cold’ tumors, in which the immune system is minimally activated, have shown poor response rates and little benefit from ICBs. In “cold” tumors, regulatory T cells (Tregs) and tumor associated macrophages (TAMs) dominate the niche, forming a suppressive TIME through inhibiting anti-tumor immune activities [1, 2]. (Fig. 1B, C). The degree of response to ICBs is based on the type of TIME. Currently, according to mouse and human data, TIME is defined as three broad classes: immune-activated, immune-defected and immune-exempted, each characterized by distinct cellular compositions. Specifically, the highest levels of Tregs form part of immune-exempted TIME inhibiting the response of immunotherapy [3].

**Fig. 2:**
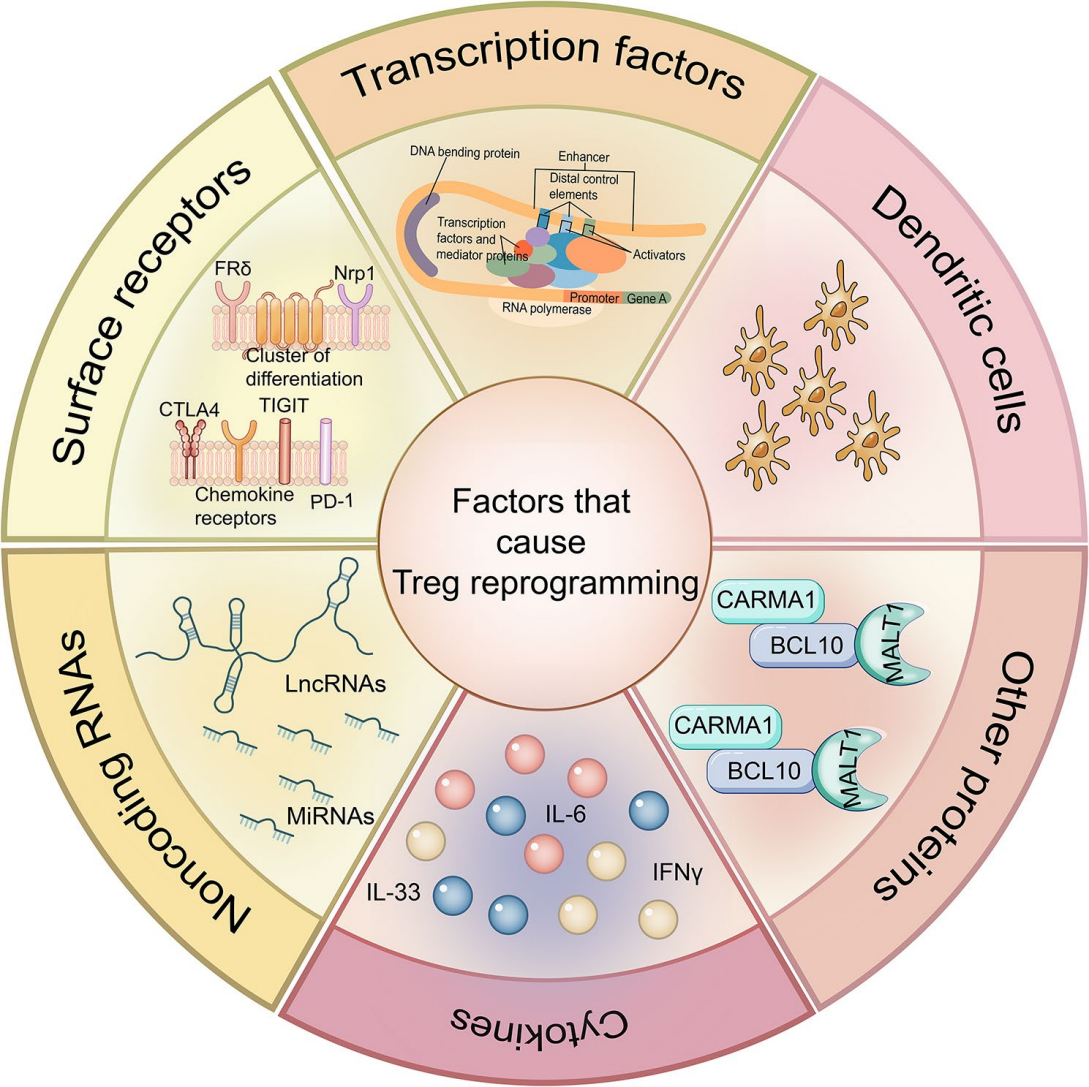
**The factors to reprogram TI-Tregs.** Treg reprogramming is facilitated by a multitude of factors, such as different transcription factors, receptors, cytokines, and others. Firstly, transcription factors induce a series of signature genes in Tregs. Their alteration causes dysregulation of gene expression and thus impairs Treg function. A portion of receptors are essential for the development, stable inhibition of Tregs, and activation or inhibition of receptors induces alterations in downstream molecular signaling thereby reprogramming Tregs. CBM is an important mediator of immune signaling, downstream of the BCR/TCR, and constitutive disruption of any of its components hinders the development of thymic Tregs, as well as the inhibitory function of mature Tregs. Non-coding RNA interactions with transcription factors define the repressive activity of Tregs. The presence of inflammatory cytokines destabilizes tumor-associated Tregs. DCs affect Treg function by triggering IL-6 expression and homologous interactions with MHC II molecules. CBM, CARMA1-BCL10-MALT1 (CBM) signalosome complex; BCR/TCR, B cell receptor/T cell receptor; DCs, Dendritic cells

Tregs, belonging to a specialized CD4^+^ T cell subpopulation, accumulate in tumors and are overactivated in diverse carcinomas to maintain immune tolerance and homeostasis and to hinder effective anti-tumor immunity [4–6]. Tregs facilitate immune escape in tumors through various mechanisms. These include the secretion of suppressor cytokines such as IL-10, TGF-β, and IL-35, as well as the inhibition of CD8^+^ T cell and dendritic cell (DC) functions via the binding of TGF-β and/or T cell receptors [7–9]. Tregs also upregulate checkpoint receptors like cytotoxic T-lymphocyte-associated protein 4 (CTLA-4), Lymphocyte Activation Gene-3 (LAG-3), and programmed death receptor 1 (PD-1), which can induce dormancy in memory T cells [10]. Additionally, they can kill effector cells through granzyme-mediated mechanisms. Tregs further inhibit the proliferation, interferon (IFN)-γ production, degranulation, and cytotoxicity of classical natural killer (NK) cells [11, 12]. This Treg-mediated inhibition is associated with the downregulation of T-cell immunoglobulin and mucin-domain containing-3 (TIM-3) and the upregulation of the inhibitory receptor PD-1, as well as IL-1R8—an IL-1 receptor family member—on classical NK cells. Furthermore, Tregs impact effector cell function by interfering with cellular metabolism, contributing to a more immunosuppressive tumor microenvironment [13]. Among Foxp3^+^ Tregs, there are two distinct subsets: central Tregs (cTregs) and effector Tregs (eTregs) [14]. Unlike cTregs, eTregs exhibit an effector-like phenotype and possess a more potent immunosuppressive capacity with significantly increased amounts of proteins that are necessary for the maintenance and suppressive activity, such as inducible co-stimulator, killer cell lectin-like receptor G1 (KLRG1), CTLA-4, and CD44^15^. The accumulation of Tregs (especially eTregs) within tumors represents a major obstacle to developing effective anti-tumor immunity. Besides, Tregs could also be divided into different subpopulations with natural Tregs (nTregs) and induced Tregs (iTregs) [16–19]. Stimulation within the tumor environment can prompt the transformation of naïve CD4^+^ T cells into ‘inducible’ Tregs [20]. As tumors progress, Tregs are recruited or induced. Specific inhibition of this subset represents a powerful approach to supporting anti-tumor response. Certain researchers have employed ablation of Foxp3^+^ Tregs to arouse tumor-antagonizing immunity, which was frustratingly devoid of clear translational potential and had diverse adverse effects [21, 22]. Therefore, expectations for Treg reprogramming which is defined as the lack of immunosuppressive function (also referred to as Treg destabilization) and/or the acquisition of pro-inflammatory or immunostimulatory properties are raised [23]. Recently, Pilato *et al*. described the reprogramming of Tregs to synthesize IFN-γ in an attempt to enhance immune checkpoint inhibitor therapy [24]. Tregs are highly plastic and heterogeneous cell populations influenced by exogenous and endogenous factors. Important endogenous factors are aberrant transcription, whereas exogenous factors are cytokines and DCs in TIME. Here, we will summarize the various factors and pathways that cause tumor-infiltrating Treg (TI-Treg) reprogramming to guide cancer immunotherapy (Fig. 2).

## Treg depletion

In contrast to reprogramming Tregs to switch their phenotype and reduce their suppressive function, Treg depletion refers to the induction of systemic or local death of Tregs by various therapeutic approaches, thereby effectively reducing or eliminating the number of Tregs. In this therapeutic strategy, anti-CD25 antibodies are typical agents used to remove Tregs in mouse models, as they can block the IL-2 signalling pathway by binding to CD25, leading to Treg death [25, 26]. Recombinant immunotoxins (RITs), such as denileukin diftitox (Ontak, a protein that binds IL-2 and diphtheria toxin) and scFv-psm-ETA, can also cause massive Treg death. However, the use of RITs to deplete Tregs has strong side effects, as this therapy also affects CD4^+^ CD25^hi^ effector T cells, further weakening the body’s anti-tumor immunity and increasing the patient’s risk of developing autoimmune diseases [25, 27].

Additionally, T cell receptor (TCR) signalling molecules in Tregs and conventional T cells (Tconv) are under different degrees of control, so another potential strategy is to target TCR signalling molecules. For example, ZAP-70 is specifically inhibited in Tregs upon TCR activation. Therefore, targeting ZAP-70 may selectively reduce TCR signalling, leading to selective death of Tregs, especially eTregs, due to apoptosis induced by signal deprivation [28]. Meanwhile, some chemotherapeutic agents may deplete Tregs by reducing their proliferation. Cyclophosphamide alkylates DNA, leading to DNA cross-linking and cell death to remove Tregs, while enhancing the efficacy of DC vaccines in melanoma or colon cancer mouse models [29, 30]. Vincristine inhibits DNA synthesis and inhibits the proliferation of IL-10-secreting Tregs in vitro, while promoting antigen-specific (CTLs) [31, 32]. Low-dose gemcitabine selectively inhibits TI-Tregs, reducing their numbers [33].

Although Treg depletion enhances the anti-tumor immune response to some extent, it has been accompanied by the major drawback of severe autoimmunity. In experimental models, systemic Treg depletion releases a strong anti-tumor response but also leads to severe morbidity. Therefore, once initiated, it seems challenging to control the autoimmune response triggered by systemic Treg depletion in patients [34, 35]. In contrast to Treg ablation, TI-Treg reprogramming may be tumor-specific and will not trigger systemic autoimmunity by interfering with the role of Tregs in maintaining peripheral homeostasis, thus reducing the incidence of immune-related adverse events. In addition, it has a number of other advantages. First, since the TCR profile of Tregs is biased towards recognition of self-antigens, reprogrammed Tregs can recognize self-antigens expressed by tumor cells without the need for new antigenic epitopes, as is the case with CTLs. Secondly, unlike CTLs, Tregs are abundant in TIME and a strategy to induce Treg conversion to effector cells would generate a large number of anti-tumor effector cells from existing Tregs that already recognize tumor self-antigens [36–38].

## Alterations in intrinsic targets in TI-Tregs lead to reprogramming

### Regulation of transcription factors in the TI-Tregs

**Fig. 3:**
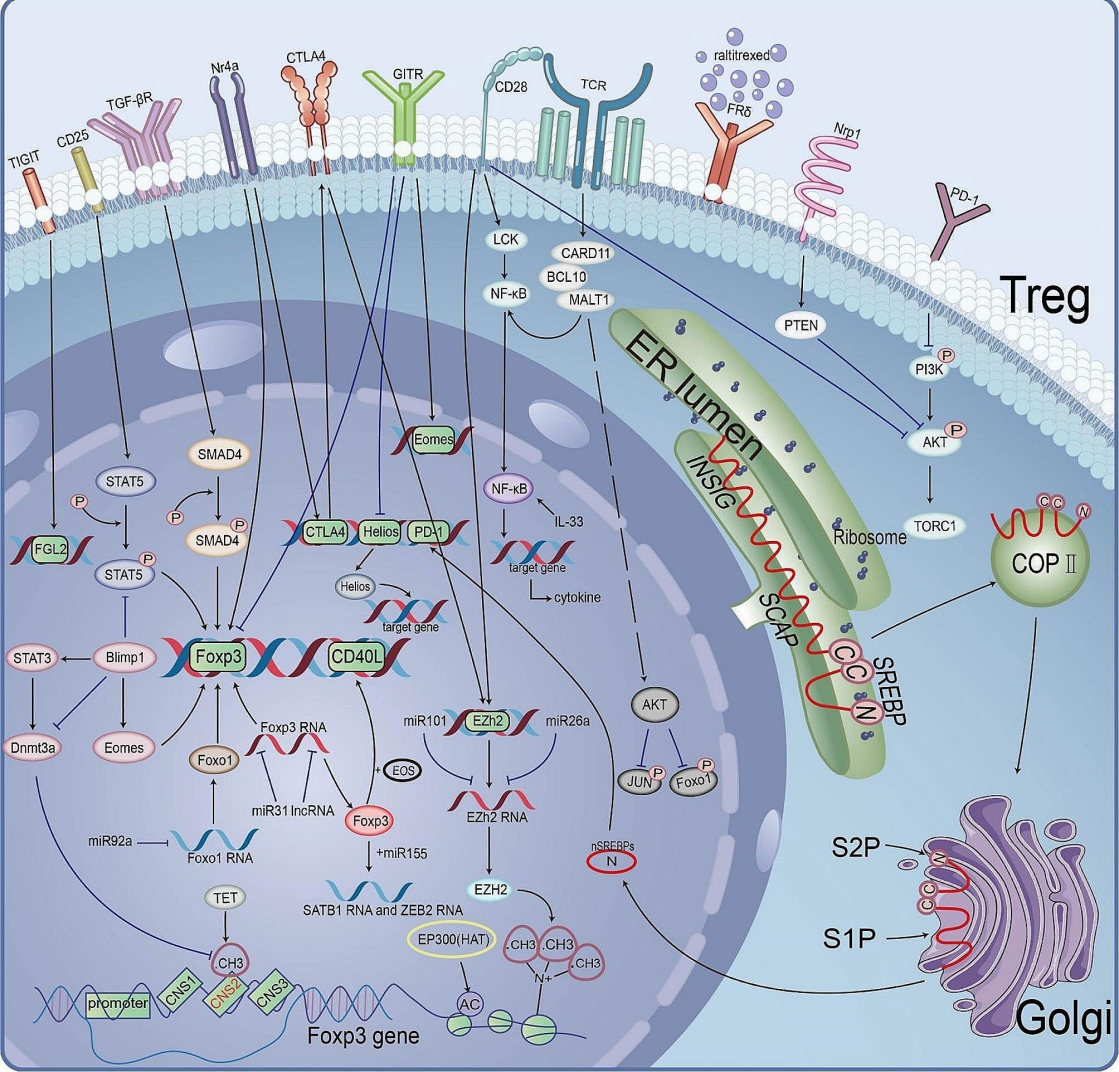
**Signals and pathways critical for the reprogramming of TI-Tregs.** Here we demarcate three interconnected nodes (transcription factors, surface receptors, constitutive intracellular signals) that together program Treg immunosuppression. Reprogramming of TI-Tregs from immunosuppressive to immune stimulatory activities is achieved by the disruption of these critical pathways. TI-Tregs, Tumor-infiltrating regulatory T cells

**Fig. 4:**
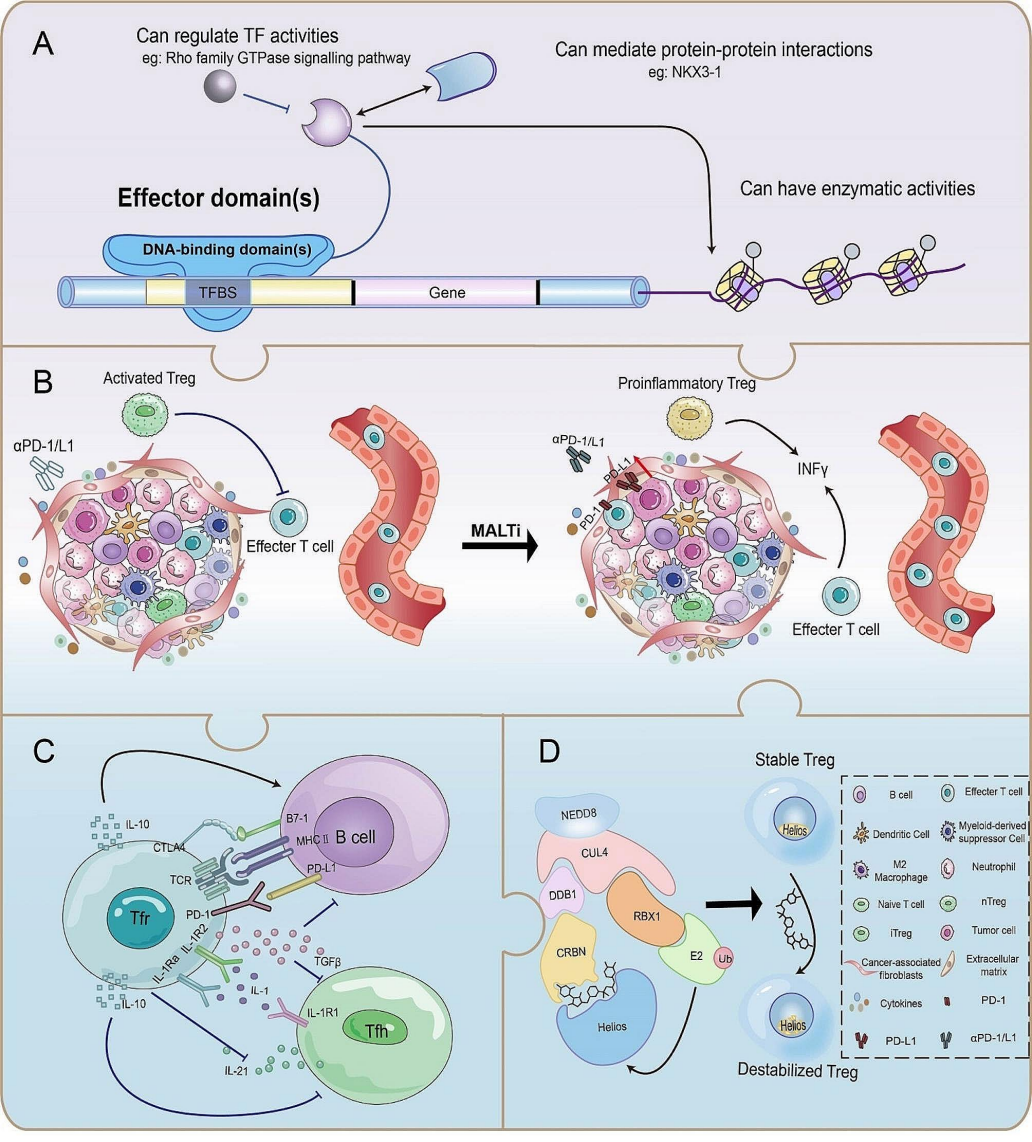
**Functions of TF; MALTi induces Treg reprogramming; adjustment functions of Tfr; targeted protein degradation.** (**A**) TFs bind DNA in a sequence-specific manner and regulate transcription. In addition, TF activity can be regulated by other signalling pathways or in interaction with other proteins to fine-tune gene expression. (**B**) Notion of MALT1 inhibitor arouses Treg reprogramming in TIME. Activated Tregs that inhabit CTLs are reprogrammed by MALT1 inhibitors (MALTi) into IFNγ-expression Tregs. Synthesized IFN-γ in an attempt to enhance local immune inflammation. Reprogrammed Tregs both enhance the recruitment and function of CTLs but also overexpress PD-L1 on cancer cells, causing acquired resistance. (**C**) Tfr cells suppresse Tfh cells and B cells through direct interaction and cytokine release. Tfr cells release TGF-β and IL-10, which inhibit the activation of Tfh cells. Additionally, Tfr cells possesse the ability to suppress Tfh cells secretion of IL-21. Moreover, the Tfr cells-expressed IL-1R2 and IL-1Ra receptors compete with the Tfh cells-surface IL-1R1 receptor for IL-1 binding. Tfr cells could build a tighter immunological synapse with B cells by producing the receptors PD-1 and CTLA4, and they could also undermine B cell activation through releasing TGF-β. Likewise, IL-10 increases B-cell activation. (**D**) A small molecule recruits the E3 ubiquitin ligase substrate receptor CRBN to Helios, thereby promoting its degradation. Disruption of Helios results in the destabilization of Tregs. TF, Transcription factors; CTLs, cytotoxic T cells; MALT1, Mucosa-associated lymphoid tissue protein 1; TIME, tumor immune microenvironment; Tfr, follicular regulatory T; Tfh, T follicular helper

Intracellular components, including various proteins, nucleic acids, cytokines, etc., are involved in regulating the stability of immunosuppressive Tregs, most notably changes in transcription factors [39] (Fig. 3). Transcription factors are DNA-binding proteins that specifically interact with the *cis-acting* elements of eukaryotic genes [40] and induce a series of signature genes in Tregs (Fig. 4A).

A multitude of studies have shown that genetic or pharmacological modulation of several transcription factors, like Eos, enhancer of zeste homolog 2 (Ezh2), and Helios, resulted in Treg reprogramming [41–43]. Meanwhile, compared with Tregs in secondary lymphoid organs and normal nonlymphoid tissues, TI-Tregs display a different transcriptional program, illustrating the viability of precisely disrupting the TI-Treg transcriptome for enhancing anti-tumor immunity [44].

#### Forkhead box protein P3 (Foxp3)

Foxp3 could trigger nTreg production in thymus and is essential for the differentiation and suppressive function in periphery. Thus, Foxp3 is regarded as the primary regulator of Treg lineage commitment [45]. Research manifested that conventional CD4^+^ T cells ectopically expressing Foxp3 would gain the same phenotype and function as Tregs [46, 47]. Foxp3 is crucial for the functional stability of Tregs [48–50]. Lahl et al. performed that the deletion of Foxp3 in scurfy Tregs abrogated individual suppressive activity, which led to a rare autoimmune disorder in humans and provoked effective anti-tumor immunity [51]. Meanwhile, Tregs with elimination of Foxp3 by a certain extent of stimulation were reprogrammed into various effector T cell lineages that secreted proinflammatory cytokines and displayed functional plasticity both in vitro and in vivo [41, 52].

Conserved non-coding sequence 2 (CNS2) elements are intronic *cis*-regulatory elements in the Foxp3 locus and composed of Treg-specific demethylation regions which loci are stably unmethylated in TI-Tregs and are vital to maintaining a stable phenotype of Tregs [53, 54]. Thus, expression of Foxp3 could be specifically regulated through epigenetic alterations, especially DNA methylation, in some of these conserved non-coding sequences (CNSs) [55]. Subsequently, some research revealed that the absence of ten-eleven translocation (TET) proteins mediating the oxidation of 5-methylcytosine to achieve DNA demethylation in CNSs wasn’t sufficient to maintain Foxp3 expression and impaired peripheral Tregs [56]. Instead, TET2 deficiency in anti-tumor effector T cells increased their potential to persist as memory cells and more effectively control cancer. Targeting TETs, therefore, presents an appealing option to judiciously stop immune suppression in TIME [57].

Foxp3 and Treg lineage stability are also impacted by the histone acetyltransferase. It has been hypothesized that lentivirus-mediated knockdown of Ep300 downregulated Foxp3 expression due to the erosion of systemic Ep300-dependent acetylation, which was partly participated by CBP/Ep300 bromodomains [58]. Utilization of inhibition by Ep300 is sufficient to reduce Foxp3 and decrease the function and homeostasis of Tregs, thereby increasing anti-tumor immunity. P300i therapy is solely applied to TI-Tregs and has no role in other immune cells, so the strategy doesn’t provoke fatal auto­immunity [21], and Ep300 could be regarded as an efficient and specific target. Moreover, there are still plenty of other factors involved in the regulation of Foxp3, such as TGF-β receptor I signaling, SMAD4, and IL-6 which upregulate Foxp3 expression. And IL-6 together with IL-1 could induce genetic reprogramming in Foxp3^+^ Tregs^52^. However, Treg lineage stability doesn’t solely depend on Foxp3 expression, as Foxp3 only triggers a part of the Treg gene signature [59].

#### B lymphocyte-induced maturation protein 1 (Blimp1)

eTregs, a specific subset of Tregs, could be distinguished from cTregs based on the differentiation in phenotype and function and are enriched in the tumors. Blimp1 encoded by Prdm1 as another essential TI-Treg regulator specifically expresses in eTregs and identifies the TI-Treg subpopulation [60]. Ablation of Blimp1 in TI-Tregs alters their lineage into effector T cells. In TI-Tregs from Prdm1^fl/fl^Foxp3^YFP−Cre^ mice, the production of IL-10 and IL-35, critical negative cytokines, was declined. This resulted in the activation of TI-CD8^ +^ T cells at the expense of Treg suppressive activity erosion. Besides the expression of IFNγ, tumor necrosis factor (TNF)-α and granzyme B was elevated, exhibiting that tumor-infiltrating Blimp1-deficient Tregs were reprogrammed [61].

The frequency of follicular regulatory T (Tfr) cells, a type of eTregs, is significantly higher and correlates with negative prognosis in numerous tumors. Meanwhile, higher tumoral Tfr cell signatures put melanoma at increased risk of metastasis [61, 62]. Tfr cells modulated by Blimp1 hinder germinal center response *via* suppressing T follicular helper (Tfh) cells and B cells and thus boost tumor growth (Fig. 4C). Blimp1-deficient Tfr cells exhibit impaired inhibitory activity and reduced production of Foxp3, CTLA-4, and other cytokines, contributing to inhibit interactions with Tfh cells and B cells and promote anti-tumor immunity, and these destabilized Tfr cells convert to Tfh-like cells [62, 63].

The regulatory details of Blimp1-induced modulation of TI-Treg stability depend on eomesodermin or directly impacting Foxp3. Through mediating Dnmt3a expression, restraining the IL-23R-STAT3 (signal transducers and activators of transduction-3) axis, or activating the CD25-STAT5 pathway, Blimp1 affects Foxp3. [64–66]. Deletion of Blimp1 contributes to the reprogramming of TI-Tregs and Tfr cells, causing them to remodel TIME, which is characterized by increased anti-tumor effector cells and enhanced anti-tumor effects [62]. The function of Blimp1 is selectively targeting tumoral Tregs since they have higher production of Blimp1 compared to Tregs in peripheral, and there are no appreciable changes in the frequencies and phenotype in splenic Tregs from Prdm1^fl/fl^Foxp3^YFP−Cre^ mice. Therefore, targeting Blimp1^+^ Tregs could produce powerful anti-tumor effects while limiting systemic toxicity, indicating a valuable target [61].

#### SREBP cleavage-activation protein (SCAP)/ sterol regulatory element-binding proteins (SREBPs)

SREBPs, which are transcription factors bound to the endoplasmic reticulum, interact with SCAP. SCAP, a protein that binds to the cell membrane, forms a complex with insulin-induced gene proteins, resulting in the inhibition of SREBP activity within the endoplasmic reticulum [67, 68]. Upon separation from insulin-induced gene proteins, SCAP/SREBPs are transported to the Golgi apparatus, where they undergo cleavage to release their transcriptionally active NH2-terminal domains. The activated NH2-terminal domains of SREBPs then enter the nucleus to initiate the transcription of target genes [69, 70].

Important functions of SREBPs have been extensively noticed, that promote lipid synthesis and uptake leading to lipid metabolism reprogramming, thereby influencing oncogenic signaling and accelerating tumor growth in malignancies [70–72]. Nevertheless, recent studies have found that SCAP/SREBP signaling is also necessary for retaining the stability and suppressive activity of Tregs in TIME. It has been reported that SREBP activity was upregulated in TI-Tregs. When SCAP was ablation, SREBPs presented inactivity and intratumoral Tregs became fragile characterized by acquisition of Foxp3 expression but abnormal IFNγ production. These demonstrated the functional state of intratumoral Tregs was SREBPs-dependent. They also found that the frequency of IFNγ^+^ Tregs was improved in the tumors of Foxp3^Cre^Scap^fl/fl^ mice compared to controls and the TIME was reshaped. There were increased CD8^+^ and CD4^+^ effector/memory T cells along with TNFα produced by conventional CD8^+^ and CD4^+^ T cells and a reduced frequency of Tregs in the remodeled TIME [73].

Specifically, SCAP/SREBPs signaling coordinates the functional integrity of Tregs through retention of lipid synthesis and inhibitory receptor signaling like PD-1 in these cells. SREBP-dependent *de novo* fatty-acid biosynthesis acts to functional maturation of Tregs. Furthermore, PD-1 function is demonstrated as a nexus with the expression of IFNγ and Phosphoinositide 3-Kinase (PI3K) signaling which impairs the functional state of Tregs, including in TIME [73, 74]. Although SREBPs are expressed in most tissues of mammals, their activity isn’t related to supporting the function of Tregs in sustaining self-tolerance at homeostasis [75]. Thus, SCAP-deficient Tregs serve as a novel therapeutic tactic provoking effective anti-tumor immunity without discernible autoimmune.

#### Helios, Eos, Nuclear receptor subfamily 4, group A (Nr4a), enhancer of zeste homolog 2 (Ezh2)

Helios (IKAROS Family Zinc Finger 2, IKZF2), the zinc finger transcription factor, which is co-expressed in Foxp3^+^Tregs, is critical for the maintenance of Treg lineage stability and suppressive activity in TIME [76], whereas Helios-deficient Tregs diminish lineage stability and acquire effector T cell function [43, 77]. The genetic basis of this Treg reprogramming is alterations in gene expression, upregulating genes as a nexus with T helper cell differentiation and effector T cell activation [43]. This phenotypic instability is primarily established in TI-Tregs but not systemic Tregs. These unstable Helios-deficient Tregs produce pro-inflammatory cytokines and enhance immune responses against cancer, providing us with an attractive tactic for inhibiting cancer progression [78]. As an intracellular transcription factor, Helios is difficult to separate from viable cell subsets for functional research and hard to drug, whereas recently it was reported that a small molecule could recruit the E3 ubiquitin ligase substrate receptor Cereblon to Helios, thereby promoting its degradation (Fig. 4D). The small molecule degraded Helios to induce an altered-Treg phenotype consistent with previously reported Ikzf2^−/−^ Tregs^4^.

Eos, also a member of the Ikaros family, in concert with Foxp3, forms a molecular complex as a paramount mediator of gene silencing arising from chromatin modifications in Tregs and thus participates in the maintenance of Treg lineage identity [79, 80]. In Tregs, silencing of Eos reprograms them to acquire immunostimulatory properties *via* increasing the expression of CD40L, and heightens immune responses against cancer. Frustratingly, there was research showing that mice with Tregs deficient in Eos exhibited side effects of organ-specific or systemic autoimmunity, which warrants vigilance [41, 80].

Nr4a factors are required to control Foxp3 and other Treg-associated genes to maintain Treg capacity of immunosuppression in TIME. Knockdown of the NR4A genes in intratumor Tregs attenuates Treg activity with the decrease of Foxp3 and CTLA-4 and develops effective T helper cell (Th) 1 and CTLs-associated anti-tumor immunity [81]. Nevertheless, it was hypothesized that these NR4A factors had resembled effects on intratumor Tregs and lymphoid Tregs, making it challenging to specifically inhibit intratumor Tregs, often endowing unintended side effects on lymphoid Tregs, but recently cyclooxygenase-2/prostaglandin E2 was identified as an attractive target due to this axis idiosyncratic enhancement of NR4A expression only in specific tumor tissues [82].

Ezh2 is preferentially expressed in TI-Tregs, where EZH2 is activated in a CD28-dependent manner and associates with Foxp3 trimethylates lysine 27 on histone H3 (H3K27me3) secondary to deposit H3K27me3 in regions of the genome that are supposed to remain silent, thereby suppressing inflammatory transcriptional programs [83]. The absence of EZH2 in Tregs drives phenotypic and functional alterations resulting in the acquisition of immunostimulatory functions with decreased production of immunosuppressive IL-10 and increased production of TNFα, IFNγ, and IL-2 that eliminate tumors and remodel the TIME, further supporting the role of EZH2 in Treg reprogramming [42]. In addition, EZH2 also promotes the transition of TAMs to the M2 phenotype and inhibits the Th1-type chemokine C-X-C Motif Chemokine Ligand 9 (CXCL9) to reduce CD8^+^ T cell infiltration, thereby reducing the anti-tumor immune response. It can also enhance the function of effector T cells and enhance the immune response through the activation of DCs. Therefore, the regulation of EZH2 to promote tumor immunity is multi-mechanism and multi-pathway [84–87].

### Regulation of surface receptors in the TI-Tregs

Compared to peripheral Tregs, several surface receptors including CTLA-4, glucocorticoid-induced tumor necrosis factor (TNF) receptor (GITR), and CD25 (IL-2 receptor α-chain) which are paramount for Tregs development, stable suppression, and cytotoxicity are upregulated in Tregs in TIME [88–90]. Blocking these key receptors on Tregs has been demonstrated to attenuate the immunosuppressive activity of intratumor Tregs and replace them with pro-inflammatory activity that enhances immune responses against cancer [42, 91]. Therefore, the identification of surface receptors involved in the reprogramming of Tregs is a promising target for cancer immunotherapy.

#### Cluster of differentiations (CDs)

CD25 is connected with the maintenance of high Foxp3 expression and lineage identity of Foxp3^+^ Tregs. Upon activation, CD25 phosphorylates and activates STAT5 which directly binds to the CNS2 intronic element, thereby affecting the expression of Foxp3, and programming immunosuppressive Tregs [78, 92]. Rech and colleagues treated human Tregs in vitro with the CD25-blocking monoclonal antibody, daclizumab, leading to selective downregulation of Foxp3, loss of inhibitory function of the Tregs, and re-secretion of IFNγ, consistent with reprogramming. In vivo, daclizumab mitigated Tregs in patients with metastatic breast cancer as expected and boosted robust CD8^+^ and CD4^+^ T cells in the absence of autoimmunity. They have validated that CD25-blocking could reprogram Tregs and promote T cell cancer immunosurveillance while avoiding autoimmunity. It should be noted, however, that most CD25 monoclonal antibodies pose an autoimmune risk in addition to daclizumab [91].

CD28 co-stimulation, an extracellular cue as the most potent secondary stimulus, plays critical roles in Tregs maintenance *via* multiple pathways, including induction of Foxp3, and regulation of the chromatin-modifying enzyme like EZH2. Noteworthy, activation of LCK (lymphocyte cell-specific protein tyrosine kinase) seems to be especially significant for Tregs. LCK is engaged in the induction and nuclear localization of nuclear factor kappa-B (NF-κB) to retain the functional integrity of Tregs in cancer [83, 93, 94]. The PI3K-AKT (protein kinase, strain AK, Thymoma)-mTOR (mammalian target of rapamycin) pathway decreases AKT activation in response to CD28 in Tregs, which hasn’t been demonstrated for its role in Treg survival and maintenance of inhibitory activity. Blocking CD28 signalling in Tregs impairs their stability and function, inhibits their ability to suppress anti-tumor immune responses, and facilitates tumor surveillance [95].

TNF receptor (TNFR) superfamily member CD357, namely GITR, is substantially increased during T-cell activation and is constitutively expressed in Tregs. GITR pathway activation abrogates tumor immune suppression through loss of Treg lineage stability [96]. Anti-GITR agonistic antibody therapy reprograms Tregs into anti-tumor Th1-like CD4^+^ T cells with the expression of IFNγ and the acquisition of cytotoxic activity against tumor cells and converts the immunosuppressive TIME into an immunostimulatory milieu causing tumor regression [37]. Mechanistically, anti-GITR causes alteration of diverse transcription factors and cytokines, for instance, downregulation of Foxp3, Helios, and IL-10 and upregulation of Eomes and IFNγ in TI-Tregs [96]. IFNγ produced by transformed Tregs may also have the additional role of driving the reprogramming of subsequent intratumor Tregs, as has been demonstrated in observations of Helios^−/−^, NRP1^−/−^, and/or CARMA^−/−^ Tregs. It has been manifested that IFNγ produced by NRP1^−/−^ Tregs is capable of reprogramming wild-type [37]. Further, mice with genetic ablation of GITR in Tregs are shown to specifically affect intratumor Tregs without peripheral autoimmunity reported [97].

#### Neuropilin-1 (Nrp-1)

Nrp1, a transmembrane glycoprotein, is expressed by ~ 90% of TI-Tregs in mouse models of cancer and acts as a co-receptor for vascular endothelial growth factors (VEGFs) isoforms mediating cell migration. Removal of Nrp-1 expression in Tregs prevents their recruitment to the tumor locus, thereby delaying the growth and progression of tumors, which could be reinstated by transferring Nrp-1^+^ Tregs adoptively from WT mice [74, 98]. Nrp1 deficiency destabilizes surrounding wild-type Tregs and loses their inhibitory activity yet retains expression of Foxp3, which is the Treg ‘fragility’. More strikingly, when they enter TIME, they are reprogrammed into IFNγ-producing cells promoting tumor clearance without autoimmune abnormalities [74]. Specifically, Nrp-1 recruits PTEN (phosphatase and tensin homolog deleted on chromosome ten) to disrupt pAKT (phosphorylated protein kinase, strain AK, Thymoma), forms the semaphorin 4 A-neuropilin 1 axis and participates in the interaction of Tregs with immature DCs, thus strengthening TI-Treg function and limiting anti-tumor immunity, while it is dispensable for Tregs to maintain immune homeostasis [74, 98–100].

#### Folate receptor delta (FRδ)

FRδ has been exploited as a biological marker for Tregs due to its exclusive expression on Tregs and oocytes [101, 102]. It has been recently discovered that the FRδ-Izumo relationship promotes immune synapses to form between Tregs and γδT cells [103]. Simultaneously, a high-affinity folate analogue, raltitrexed, has been identified to specifically bind to FRδ in tumors other than peripheral blood and healthy tissues. This specific ligand has been used to selectively target therapeutic agents (imaging agents, immune activators, and immunosuppressants) to Tregs in murine tumor xenografts. For instance, delivery of a TLR7 agonist to Tregs has been shown to reprogram the TIME into a state of lower immune suppression, resulting in approximately 40–80% reduction in tumor development without causing appreciable systemic damage [104].

#### PD-1

Research has shown that the expression of PD-1 on Tregs promotes the homeostasis and functionality of Tregs. In mice with conditional deletion of PD-1, the function of TI-Tregs declines, and their numbers are reduced. Meanwhile, Foxp3 tracking experiments indicate that the expression of PD-1 is crucial for the stability of Foxp3 expression in TI-Tregs, as Foxp3 expression is downregulated. Single-cell analyses reveal that PD-1 signalling enhances lipid metabolism, proliferation, and inhibitory pathways in TI-Tregs. Foxp3 has been reported to regulate the suppressive function of Tregs by suppressing glycolysis and enhancing oxidative phosphorylation. These findings suggest that the absence or inhibition of PD-1 can weaken the lineage stability and metabolic adaptability of Tregs in TIME by reducing Foxp3 expression, thereby enhancing anti-tumor immunity [105–107]. However, the function of PD-1 in TI-Tregs remains controversial. In EAE mice, the expression of PD-1 has been reported to inhibit the immunosuppressive function of Tregs. Furthermore, a higher incidence of progressive disease has been observed in some cancer patients receiving anti-PD-1 treatment [108, 109]. Another study reported that excessive progression was unrelated to the activity of TI-Tregs but was associated with the pre-treatment frequency of CD39^+^ CD8^+^ T cells [110]. Thus, further research is necessary to understand how PD-1 functions in Tregs depending on the situation.

#### CTLA-4

CTLA-4 is a homolog of CD28 and is constitutively expressed on the surface of Tregs. CTLA-4 competes with CD28 for binding to CD80/CD86 on antigen-presenting cells (APCs), thereby inhibiting CD28-mediated co-stimulation of T cells [88, 111]. According to certain research, CTLA-4 can reduce the immunosuppressive activity of Tregs. Immune checkpoint blockade with anti-CTLA-4 significantly increases the expression of EZH2 in the TIME and may enhance the suppressive activity of TI-Tregs [42]. Anti-CTLA-4 monoclonal antibodies can also deplete Tregs, and in cancer patients, there is a strong correlation between the clinical efficacy of Ipilimumab and the reduction of Treg numbers in tumor tissues [28]. The effects of CTLA-4 blockade on Tregs are complex, and different antibodies may have distinct mechanisms of action (for instance, either depleting Tregs or functionally inhibiting them without depletion) [112, 113]. Therefore, while CTLA-4 is an attractive target for destabilizing and reprogramming Tregs, its effects still require further investigation.

#### T cell immunoglobulin and ITIM domain (TIGIT)

TIGIT is an inhibitory receptor expressed on lymphocytes, primarily on activated T cells, NK cells, and Tregs. It has recently emerged as a major new target in cancer immunotherapy [114]. TIGIT competes with CD226 for interaction with CD112 and CD155 (PVR). The CD155/TIGIT signaling pathway exerts immunosuppressive effects by exacerbating cellular exhaustion, leading to tumor immune evasion. TIGIT signalling has the potential to enhance the suppressive function of Tregs [115]. Agonistic monoclonal antibodies to human TIGIT (hu-TIGIT) have been reported to induce Treg effector molecule fibrinogen-like protein 2. These antibodies are an agonistic monoclonal antibody targeting TIGIT that selectively and potently suppresses T follicular helper (Tfh) cells while promoting the suppressive function of Tregs [116]. Besides, TI-Tregs respond to Fc-active anti-TIGIT antibodies and downregulate immunosuppressive gene programs.

Conversely, other studies indicate that increased expression of TIGIT on Tregs is associated with decreased Treg function. Aspirin has shown significant anti-colorectal cancer effects in mice. During related mechanistic studies, it was found that there were more CD155 tumor cells and CD4^+^ CD25^+^ Tregs, with increased TIGIT levels following aspirin treatment. The high expression of TIGIT reduces the functionality of Tregs, thereby decreasing their immunosuppressive effects and promoting the anti-tumor actions of other immune cells in the colorectal cancer (CRC) immunological microenvironment [117].

#### Chemokine receptors

After activation, Tregs specific express the accompanying chemokine receptors, such as the chemokine receptors C-C Motif Chemokine Receptor (CCR) 4 and CCR8. Focusing on specific subsets of TI-Tregs by evaluating their chemokine receptor expression is currently a key area of research for cancer treatment [118].

CCR4 and CCR8 have recently been identified as being more selectively expressed on tumor-reactive eTregs [90, 119, 120]. The expression of CCR4 demonstrates an enhanced ability to suppress effector T cells. Functionally, CCR4^+^ Tregs are more immunosuppressive compared to CCR4^−^ Tregs. Pharmacological antagonism of CCR4 resulted in a decreased frequency of Tregs and hindered the maintenance of the TI-Treg pool [121].Moreover, antagonizing CCR4 prevented the accumulation of macrophages and myeloid-derived suppressor cells (MDSCs), which are two other suppressive cell types that contribute to immune evasion. This positions CCR4 antagonism as a potentially more versatile strategy for anti-tumor immunotherapy [122, 123].

Besides, CCR4 ligands are found to be upregulated in tumors following treatment with checkpoint inhibitors. The blockade of CCR4 demonstrated a synergistic anti-tumor effect in conjunction with these immunomodulatory agents [121].

The results of a single-cell analysis of TI-Treg revealed that multiclonal Tregs predominantly express the chemokine receptor CCR8, which correlates with enhanced activation and suppressive capabilities. Compared to CCR8^–^TI-Tregs, CCR8^+^TI-Tregs exhibit enhanced activation, greater suppressive characteristics, and increased stability, as demonstrated by elevated expression of activation and suppression markers and Treg-specific DNA hypomethylation. Targeting CCR8 with anti-CCR8 mAb not only reduced multiclonal Tregs specifically in tumor tissues but also evoked strong anti-tumor immune responses without causing harmful autoimmunity [34, 90, 120, 124]. Ultimately, CXCR3 serves as the key chemokine receptor for Th1 cells, while CCR6 is characteristic of Th17-like Tregs. Targeting these receptors may similarly achieve tumor-modulating effects, although this warrants further investigation [125, 126].

### Regulation of other constitutive intracellular signals in the TI-Tregs

In addition to cell surface-specific receptors and intracellular transcription factors, there are miscellaneous other intracellular signals involved in the regulation of Treg reprogramming.

#### CARMA1-BCL10-MALT1 (CBM) signalosome complex

CBM is ubiquitously and heterogeneously expressed in almost all human tissues and cells and is an essential mediator of immune signaling. In lymphocytes, CBM signalosome assembled by CARD11 (caspase recruitment domain 11, also known as CARMA1), BCL10 (B cell lymphoma/leukemia protein10) and MALT1 (Mucosa-­associated lymphoid tissue protein 1, also termed paracaspase 1), mediates the activation of NF-κB and Jun N-­terminal kinase (JNK) on ligand binding to T/B cell receptor signalling. This alters relevant cellular activation, differentiation, and effector function [127, 128]. CBM is necessary for nTreg development, iTreg induction, and the conversion of cTregs to eTregs under steady-state conditions, as well as crucial for accessing the capacity of suppression of Tregs [127, 129]. Constitutive genetic disruptions in its any component could hurdle thymic Treg development, abrogate inhibitory function of mature Tregs, and a majority of TI-Tregs produce IFNγ, rising to decelerate tumor growth [24, 127].

In inflammatory conditions, Treg-specific ablation of the scaffold protein CARD11, destabilizes Tregs in tumor tissues and secretes IFNγ to decline tumor growth, revealing its primary role in the depression of anti-tumor effects. In this condition, only eTregs express Th1 lineage-defining transcription factor undergoing a lineage identity transition, and these shift Tregs maintain Foxp3 expression [24]. Whereas in the context of non-inflammatory lack of CARD11 doesn’t cause the above-mentioned change. The regulatory mechanisms of CARD11 deficiency include not only the failure of NF-κB signaling [130]. but also reduced expression and TCR-induced phosphorylation of the JUN. Additionally, there are alterations in the phosphorylation of Forkhead box O1 (Foxo1) [131]. Notably, selective ablation of both alleles of CARD11 in Tregs endowed fatal immune pathology, while partial deletion of CARD11 in only a fraction of Tregs to provoke their production of the IFNγ and TNF was sufficient to generate anti-tumor effects and avoid detectable immune pathology. Heterozygous CARD11-damaged mice were healthy and had a normal lifespan, but transplant cancers grew more slowly in these animals. Interestingly, tumor cells raised PD-L1 production, which widely validated activation of adaptive immune resistance. Consequently, tumors ineffective on anti-PD-1 monotherapy were rejected when PD-1 inhibition and CARD11 deletion were combined [24].

Activated CARD11 interacts with BCL10 and promotes BCL10 formation of macromolecular filaments, providing a large scaffold for MALT1 binding and activation. The formation of BCL10 filaments is also essential for amplifying BCR/TCR signalling and the robust activation of downstream NF-κB [132]. Studies in BCL10 conditional knockout mice have demonstrated that deletion of BCL10 in immature Tregs decreases Treg populations, whereas specific deletion of BCL10 in mature Tregs impairs the inhibitory function of mature Tregs, leading to their transformation into IFNγ-producing pro-inflammatory cells with much higher expression of transcription factor T-bet and hypoxia-inducible factor-1α (HIF-1α) [15].

Similar anti-tumor activity is also observed following ablation of MALT1 in Tregs which is the molecular scaffolding and enzymatic paracaspase domain of the CBM complex that further activates the downstream functions of diverse effector molecules [132]. Counterintuitively, neither TNF receptor-associated factor 6 (TRAF6) deficiency nor destruction of MALT1-TRAF6 interaction influences NF-κB activation in response to inflammatory TNFα, revealing MALT1 protease activation primarily drives NF-κB signalling which controls the development and suppressive functions of Tregs [133]. MALT1 protease activity is required for Tregs to be susceptible to innate immunological stimulation and maintains high CTLA-4 levels on Tregs, thus MALT1 has a pivotal function in balancing thymic and peripheral tolerance. MALT1 paracaspase dysregulation selectively reprograms immunosuppressive Tregs to a pro-inflammatory fragile state in TIME and gives rise to ‘scurfy-like’ autoimmune syndromes [134] (Fig. 4B). Therefore, when reprogramming TI-Tregs with MALT1 inhibitors for the treatment of solid tumors, attention for occurrence of autoimmune toxicity is warrant [135].

#### Noncoding RNAs

Tregs are also regulated by abnormal molecular expression of noncoding RNAs, including microRNAs (miRNAs/miRs) and long noncoding RNAs (lncRNAs), which don’t translate proteins but instead interact with transcription factors defining Treg function and improving anti-tumor responses [136–138]. MiRNAs interact with multiple transcription factors. Firstly, they interact extensively with Foxp3. MiRNA profiles could reinforce Foxp3 expression maintaining Treg suppressive capacity. Impairment of miRNA function downregulates the level of microRNA-mediated Foxp3 and develops fatal systemic autoimmune disease. Furthermore, miRNAs are also direct or indirect targets of Foxp3 which conjugates vicinity of miRNA-encoding intergenic sequences in Tregs. In addition, miRNAs such as miR-155 cooperate with Foxp3 to coordinate the regulation of other key genes in Tregs, including special AT-rich sequence binding protein 1 (SATB1) and Zinc finger E-box binding homeobox 2 (ZEB2) [139–141]. This interaction could be observed in investigations of miR-31. Secondly, miRNAs mediate the transcription factor Foxo1, which binds directly to the Foxp3 motif to support Foxp3 expression and also acts on the Ifng motif to repress Ifng expression [142, 143], playing a vital role in early Treg lineage stability. For example, miR-92a abrogates Treg homeostasis and function and facilitates Tregs acquiring an inflammatory phenotype by repressing Foxo1. Research has shown that exposure of wild-type Tregs to inflammatory environments results in an increase in IL-17 A or IFN-γ. In contrast, miR-92a^−/−^ Tregs continue relatively resistant to these inflammatory cytokine-mediated changes both in vivo and in vitro. In the meantime, these inflammatory stimuli have caused upregulation of miR-92a correlated with decreased expression of the miR-92a target Foxo1 in WT Tregs. These results suggest that miR-92a targeting Foxo1 boosts the procurement of inflammatory Treg phenotype and destroys Treg suppressive function [136].

Afterward, there are also other sorts of miRNAs involved in the regulation of Tregs. For example, miR-101 and miR-26a regulate EZH2, which is relevant for Foxp3 to retain suppressive capacity of nTreg in mice. miR-155 significantly decreases the abundance of Tregs [144–146]. Foxp3^Cre^miR-142^fl/fl^ mice negatively regulate the frequencies and suppressor function of Tregs [147]. Loss of miR-181a/b-1 increases Treg inhibition function and negatively associates with the expression of CTLA-4 protein in the thymus and peripheral Tregs. Similarly, the miR-17-92-deficient Tregs lead to tumor immune evasion [148, 149]. Distinct miRNA profiles of human nTregs include miRNAs that positively regulate the expression of key Treg genes, such as miR-21 [150], 125a, 142 [151], 146a, 181c, and 374 [152], as well as negative regulator miRNAs, such as miR31 miR-155 miR-181a/b-1[153, 154]. These miRNAs comprise part of a network of regulatory factors in Tregs (Table 1) [153, 154].

**Table 1:** Noncoding RNAs influence the stability of Tregs

Noncoding RNAs	Function	References
MiR31	1. Target the 3’ UTR of Foxp3 mRNA to negatively regulate Foxp3 expression. 2. Discover a target sequence for Foxp3 within the mouse miR-31 encoding gene’s promoter region, indicating that Foxp3 may directly target miR-31.	[150, 196, 197]
MiR92a	1. Abrogate Treg homeostasis and function and facilitate Tregs acquiring an inflammatory phenotype by repressing Foxo1.	[136]
MiR-101 and MiR-26a	1. Regulate EZH2 to stabilize Foxp3.	[144]
MiR-155	1. Decrease the abundance of Tregs. 2. Cooperate with Foxp3 to regulate special AT-rich sequence binding protein 1 (SATB1) and Zinc finger E-box binding homeobox 2 (ZEB2).	[144]
MiR-142	1. Impact the frequencies and suppressor function of Tregs	[147]

LncRNAs are untranslated transcripts longer than 200 bp that are subtle regulators of gene expression through interaction with mRNAs and chromatin [155, 156]. The important role of lncRNA in Treg commitment has been proven dependent on binding to Foxp3 conserved noncoding elements and influencing its expression. Silencing lncRNA leads to the downregulation of Foxp3. This could be a necessary condition for Treg transcriptional reprogramming in response to external stimuli like decreased IL-2 signaling, supporting the fact that transcriptional networks could undergo very little alterations to generate plasticity [157, 158].

#### IL-33

Intranuclear IL-33 affects Tregs’ transcriptional profile and determines their activity in the anti-tumor immune response. IL-33-deficient Tregs exhibit compromised suppressive capabilities and aid in the elimination of tumors as well as the development of strong anti-tumor immunity. Ablation of IL-33 reprograms Tregs to upregulate IFNγ expression and maintain Foxp3 expression, consistent with a “fragile” phenotype, which exhibits reduced suppressive function in vivo, thereby accelerating tumor regression [159, 160]. The molecular events that elevate IFNγ production depend on the NF-κB-T-bet–IFNγ axis in IL-33-deficient Tregs. Gene enrichment analysis showed that Tbx21 which codes for T-bet and is an essential hub in the Ifng gene regulation network was differentially expressed in IL-33^−/−^Tregs comparison with control. In support, TI-Tregs from Foxp3^Cre^IL33^fl/fl^ mice displayed significantly more T-bet expression than control Foxp3^Cre^ mice and increased accessibility of the site of T-bet binding in the regulatory region of the Ifng locus. What’s more, the expression of T-bet is regulated by intracellular IL-33-regulated NF-κB signaling which inhibition significantly downregulates T-bet and IFNγ expression. Combinedly, these findings claim that in IL-33^−/−^ mice TI-Treg reprogramming is in an NF-κB-T-bet-IFNγ manner [161, 162].

## Alterations in extracellular targets in TIME induce TI-Treg reprogramming

### Regulation of inflammatory factors drives Treg reprogramming in TIME

At sites of inflammation, Tregs become dysfunctional and undergo rapid reprogramming into Th1 or Th17 expressing inflammatory cytokines [136]. Both in vitro and in vivo, exposure to IL-6 changes the fate of Tregs transdifferentiate into Th17 expressing Th17-specific genes and downregulation of Foxp3 [163]. In anti-tumor immunotherapy, blocking the IL-6 receptor (IL-6R) on Tregs results in the loss of the ability to destabilize and reprogram Tregs [164, 165]. Early in vitro studies found Foxp3 expression was decreased in activated Tregs with the presence of IL-6, which is mediated by STAT3, RAR-related orphan nuclear receptor (ROR) α, and RORγt [163, 166]. IL-6 facilitates STAT3, a critical component of signal transduction, inducing a series of miRNAs that cause IL-17-producing, diminishing Foxp3 expression, and causing RORγt to dissociate from Foxp3. Secretion of IL-17 and IL-17 F is dependent on RORα and RORγt. Subsequently, the generation of IL-17-secreting Tregs and Th17 is induced [52, 163]. It is of note that IL-6 appears to also drive Eos downregulation, which has been proven to be an important driver of Treg reprogramming. Moreover, the addition of high concentrations of neutralizing anti-IL-6 to the co-cultures overcomes the lack of Eos and prevents functional reprogramming [163].

IFNγ is also critical for Treg dysfunction. Its ablation restores IL33^−/−^Treg suppressive properties and IFNγ produced by transformed Tregs, as mentioned above, may also have an additional role of driving subsequent intratumoral Treg reprogramming. The instability of Tregs was discovered to require the expression of the IFNγ receptor (IFNγR1), indicating the possibility of an IFNγ-driven autocrine or paracrine loop [37]. Notably, deletion of IFNγR renders Tregs unresponsive to this destabilizing IFNγ, and then traditional PD-1 checkpoint blockade immunotherapy fails [74]. Thus, pro-inflammatory cytokines appear to convey important signals that disrupt the stability of tumor-associated Tregs. And, among the massive models tested, this successful immunotherapy requires an unstable effect on Tregs.

### DCs involve in TI-Treg reprogramming

**Fig. 5:**
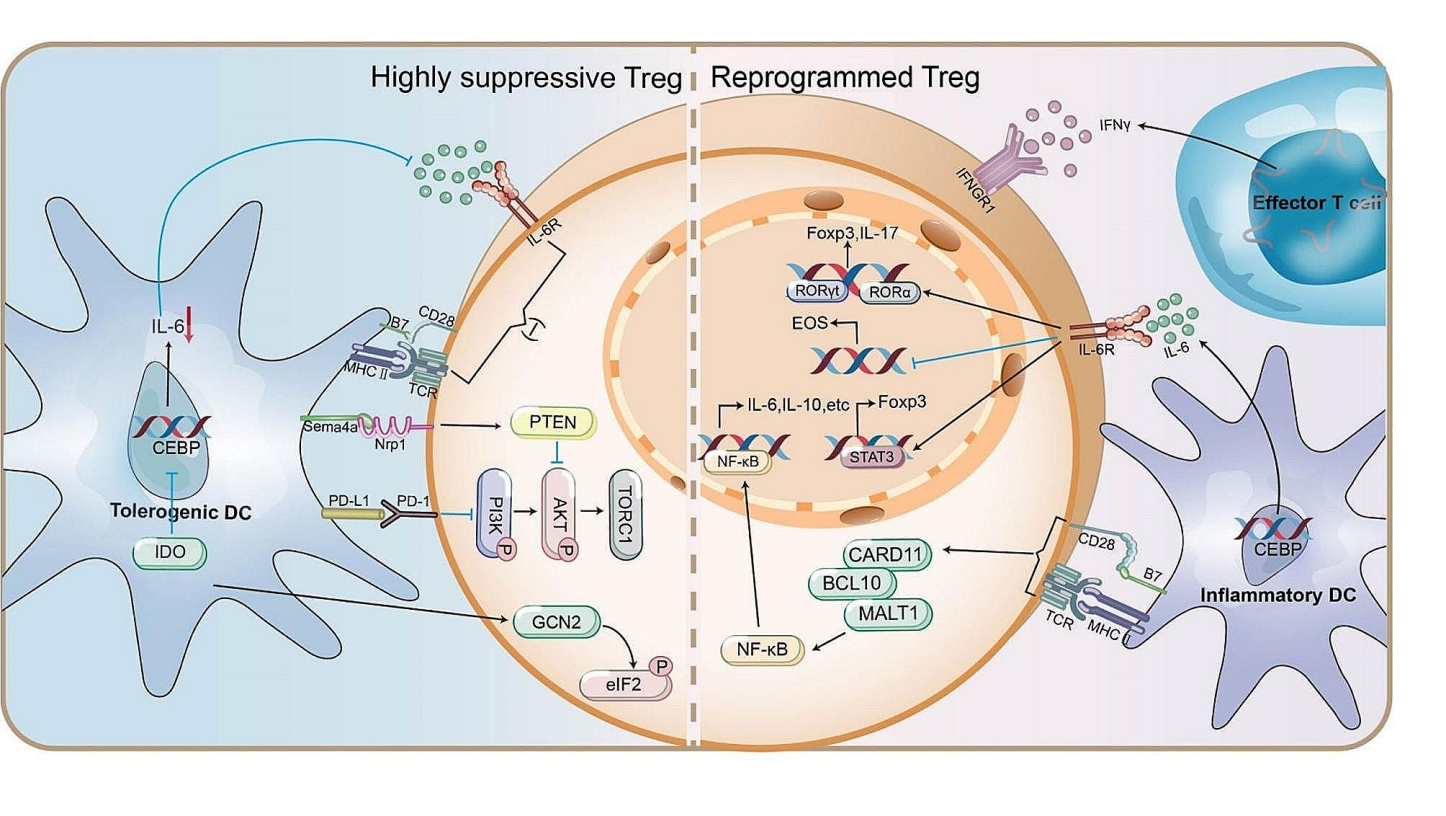
**Effects of inflammatory factors and DCs on Treg stability.** Two key pro-inflammatory cytokines, IL6 and IFNγ, deliver important signals that destabilize TI-Tregs. Inflammatory DCs participate in the reprogramming of Tregs through triggering IL-6 expression and cognating interaction with Tregs via MHC II. Tolerogenic tumor-associated DCs express IDO, PD-L1, or semaphorin-4a (the ligand for Nrp1) as well as restrain IL-6 expression and interaction with Tregs to resist the inflammation-induced reprogramming of Tregs in vivo. TI-Tregs, Tumor-infiltrating regulatory T cells; DCs, Dendritic cells; IDO, indoleamine 2,3 dioxygenase; Nrp1, Neuropilin-1

Proverbially, TIME contains a variety of immune cells with a wide range of interactions that are capable of recruitment, inducement, and maintenance of Tregs. Inflammatory DCs are required for Treg reprogramming, driving a substantial fraction loss of suppressor phenotype and acquisition of pro-inflammatory phenotype. Sharma and colleagues used a co-culture model in vitro to validate that reprogramming required activated DCs rather than resting DCs to trigger IL-6 expression and cognate interaction with Tregs *via* MHC II [163]. Besides, tolerogenic tumor-associated DCs express IDO (indoleamine 2,3 dioxygenase), PD-L1, or semaphorin-4a (the ligand for Nrp1) to resist the inflammation-induced reprogramming of Tregs in vivo. Mechanisms by which IDO affects Treg reprogramming include blocking IL-6 production through effects on NF-IL6 (CEBP) or activating the General control nonderepressible 2 (GCN2) kinase pathway in Tregs phosphorylating the ribosomal translation factor eukaryotic Initiation Factor 2 (eIF2) to alter ribosomal translation of mRNA species [20] (Fig. 5). This event also occurs in tumor-draining LNs, as before, Sharma used plasmacytoid DCs (pDCs) expressing IDO from tumor-draining LNs co-cultured with Tregs in vitro to demonstrate [163]. Interestingly, IDO-expressing macrophages have been found in carcinomatous ascites, so there may be more than one type of antigen-presenting cell capable of expressing IDO in human tumors and tumor-draining LN and they may also perform a function to influence Treg reprogramming, which remains to be established [20].

## Translation of results: Treg reprogramming in therapy

### Clinical strategies of Treg reprogramming for oncology treatment

The instability of intratumoral Treg populations alters the immune response to tumor cells, with profound therapeutic implications. A wide variety of components are involved in the Treg reprogramming process, which we have summarized here (Table 2). Based on the above understanding, one possible clinical strategy that has been proposed is to block one of the known Treg-stabilizing signals in Tregs or TIME, such as MALT1, or Helios. Pleasantly, two clinical candidates of potent and selective allosteric MALT1 inhibitors, JNJ-67,856,633 [167] and MPT-0118, have been developed based on structure-guided drug research. Clinical trials adjudicate the role of MPT-0118 as a single agent or in combination with PD-1 blockade in Treg phenotypic transformation by a tumor-cell extrinsic pathway [168]. Considering the dual effects of MALT1 in immune TIME and cancer cells, that is, inhibiting MALT1 protein activity affects tumor cell proliferation, survival, and invasion while reprogramming activates Tregs to secrete IFNγ, the use of MALT1 inhibitors could appropriately multiply the anti-tumor immunity, improve ICT response in solid cancers, and reduce tumor cell growth and infiltration with less effort. It has reported the discovery of NVP-DKY709, a selective molecular glue degrader of IKZF2(Helios) that preserves IKZF1/3. Treatment with NVP-DKY709 reduced the inhibitory activity of human Tregs and rescued cytokine production in depleted T effector cells. Its role of delayed tumor growth in mice with a humanized immune system and cynomolgus monkeys has been confirmed. NVP-DKY709 is being investigated in the clinic as an immune enhancer for cancer immunotherapy [169]. Meanwhile, pharmacological Helios degraders VLV1, and VLV2, novel small molecule ligands of CRBN, also disrupt phenotypic stability and reduce the inhibitory activity of human Tregs in vitro, establishing a route towards Helios-targeting therapeutics. Encouragingly, targeted protein degradation has been found in the R&D process, which is the development pathway for emerging small molecules used to induce ubiquitination and subsequent proteasomal degradation of target proteins, which have the potential to expand the druggable proteome [4].

**Table 2:** Representative factors to reprogram TI-Tregs.

Target	Category	Description	Mechanisims of action	References
Foxp3	Transcription factors	1. Forkhead/winged-helix family of transcriptional regulators. 2. Trigger the production of tTregs and regulate the differentiation and inhibitory function of pTregs. 3. Trigger the transcription of Treg characteristic genes and maintain the stable phenotype of Tregs.	-	[45, 59, 182–184]
Blimp1	Transcription factors	1. The zinc finger protein family and repressor of beta-interferon gene expression. 2. Involved in the production of IL-10 and IL-35. 3. Regulate Tfr cells and deletion of Blimp1 facilitates the reprogramming of Tfr cells. 4. Elimination of Blimp1 in TI-Tregs changes their lineage to Teff.	1. Depend on eomesodermin or directly impact Foxp3 through mediating Dnmt3a expression to maintain the methylation of CNS2. 2. Restrain the IL-23R-STAT3 axis. 3. Activate the CD25-STAT5 pathway.	[61–66, 185]
SCAP/SREBP	Transcription factors	1. In the presence of cholesterol, SCAP binds and hydrolyzes to activate SREBPs. 2. SREBPs promote lipid synthesis and uptake, leading to lipid metabolism reprogramming. 3. Maintain the stability and inhibitory activity of TI-Tregs. 4. Deletion of SCAP leads to an increase in the frequency of IFNγ ^+^ Tregs.	1. Induce de novo fatty-acid biosynthesis to boost functional maturation of Tregs. 2. Sustain PD-1 function to maintain expression of IFNγ.	[70–74]
Helios	Transcription factors	1. The Ikaros family of zinc-finger proteins. 2. Unstable Helios-deficient Tregs produce pro-inflammatory cytokines and enhance immune responses against cancer.	1. Stabilize the phenotype of Tregs, possibly via signal transducer and activator of transcription 5 (STAT5)–mediated signaling and prevention of interleukin-2 (IL-2) production in Tregs by epigenetic silencing.	[78, 186, 187]
Eos	Transcription factors	1. The Ikaros family. 2. In concert with Foxp3, form a molecular complex to silence genes. 3. Participate in the maintenance of Treg lineage identity.	-	[79, 80]
Nr4a	Transcription factors	1. The steroid-thyroid hormone-retinoid receptor superfamily. 2. Promote the development of Tregs by cooperating with other Treg developmental mechanisms. 3. Exhaustion of NR4A attenuates Treg activity with the decrease of Foxp3 and CTLA-4 and develops Th1 and CTLs-associated anti-tumor immunity.	1. Control Foxp3 and other Treg-associated genes.	[81, 188, 189]
Ezh2	Transcription factors	1. The Polycomb-group (PcG) family. 2. The absence of EZH2 in Tregs drives the acquisition of immunostimulatory functions with decreased production of immunosuppressive IL-10 and increased production of TNFα, IFNγ, and IL-2.	1. Be activated in a CD28-dependent manner and participate in Foxp3 trimethylates lysine 27 on histone H3 secondary to remain genome silent.	[42, 83, 184, 190–192]
CD25	The IL-2 receptor α chain	1. Together with the common beta (IL2RB) chains and gamma chain (IL2RG), constitute the high-affinity IL2 receptor. 2. CD25-blocking leads to selective downregulation of Foxp3, loss of inhibitory function of the Tregs, and re-secretion of IFNγ.	1. Phosphorylate and activate STAT5 which directly binds to the CNS2 intronic element, affecting the expression of Foxp3, and programming immunosuppressive Tregs.	[78, 91, 92]
CD28	Receptors for costimulatory molecules	1. Participate in T-cell proliferation and survival, cytokine production, and T-helper type-2 development.	1. Induce Foxp3, and regulate the chromatin-modifying enzyme like EZH2.	[83, 93]
GITR	Receptors	1. The TNF receptor (TNFR) superfamily. 2. Play a key role in dominant immunological self-tolerance maintained by CD25(+) CD4(+) Tregs. 3. Anti-GITR agonistic antibody therapy reprograms Tregs into anti-tumor Th1-like CD4 ^+^ T cells with the expression of IFNγ.	1. Alter diverse transcription factors and cytokines, for instance, downregulation of Foxp3, Helios, and IL-10 and upregulation of Eomes and IFNγ in TI-Tregs.	[37, 96, 193]
Nrp1	Receptors	1. The co-receptor for vascular endothelial growth factors isoforms mediating cell migration. 2. Nrp1-deficient destabilizes Tregs and contributes to Treg ‘fragility’. 3. When ‘fragility’ Tregs enter TIME, they are reprogrammed into IFNγ-producing cells promoting tumor clearance.	1. Recruit PTEN to disrupt pAKT. 2. Form the semaphorin 4 A-neuropilin 1 axis. 3. Participate in the interaction of Tregs with immature DCs.	[74, 98–100]
FRδ	Receptors	1. Predict to be located in extracellular region and plasma membrane. 2. Promote immune synapses to form between Tregs and γδT cells.	-	[104]
PD-1	Receptors	1. An important immunosuppressive molecule. 2. The function of PD-1 in TI-Tregs remains controversial.	1. Reduce Foxp3 expression. 2. As a nexus with the expression of IFNγ and Phosphoinositide 3-Kinase (PI3K) signaling	[105–107]
CTLA-4	Receptors	1. Anti-CTLA-4 enhances the suppressive activity of TI-Tregs and depletes the number of Tregs.	1. Increase the expression of EZH2.	[42, 88]
TIGIT	Receptors	1. Expressed as an inhibitory receptor on lymphocytes. 2. Enhance the suppressive function of Tregs.	1. Induce Treg effector molecule fibrinogen-like protein 2.	[114–116]
CCR4	Receptors	1. CCR4^+^ Tregs are more immunosuppressive compared to CCR4^-^ Tregs. 2. Prevent the accumulation of Tregs.	-	[121, 194]
CCR8	Receptors	1. Exhibit enhanced activation, greater suppressive characteristics, and increased stability.	-	[34, 90, 120]
CBM signalosome complex	Immunomodulatory protein complex	1. Assembled by CARD11, BCL10 and MALT1. 2. Promote nTreg development, iTreg induction, and the conversion of cTregs to eTregs under steady-state conditions, as well as accessing the capacity of suppression of Tregs. 3. Constitutive genetic disruptions in its any components hurdle tTreg development, abrogate inhibitory function of mature Tregs, and a majority of TI-Tregs produce IFNγ.	1. Trigger NF-kappaB signaling, Jun N-­terminal kinase and lymphoctye activation following antigen-receptor stimulation. 2. Possibly regulate Akt activity.	[127–129]
MiRNAs	Noncoding RNAs	1. Reinforce Foxp3 expression to maintain Treg suppressive capacity.	1. Bind the 3’-untranslated regions of target mRNAs to increase mRNA digestion or block mRNA translation and thus, involve in post-transcriptional regulation of gene expression. 2. Mediate the transcription factor Foxo1, which binds directly to the Foxp3 motif to support Foxp3 expression and also act on the Ifng motif to repress Ifng expression.	[136]
lncRNAs	Long noncoding RNAs	1. Silence lncRNA leading to the downregulation of Foxp3.	1. Interact with mRNAs and chromatin to regulate gene expression.	[155]
IL-33	Pro-inflammatory cytokine	1. Affect Tregs’ transcriptional profile and determine their activity. 2. Ablation of IL-33 reprograms Tregs to upregulate IFNγ expression and maintain Foxp3 expression, consistent with a “fragile” phenotype.	1. Depend on the NF-κB-T-bet–IFNγ axis.	[161, 195]
IL-6	Pro-inflammatory cytokine	1. Change the fate of Tregs transdifferentiate into Th17 expressing Th17-specific genes and downregulation of Foxp3.	1. Reduction of Foxp3 is mediated by STAT3, RAR-related orphan nuclear receptor (ROR) α, and RORγt. 2. Induce a series of miRNAs causing IL-17-producing, diminishing Foxp3 expression, and causing RORγt to dissociate from Foxp3.	[163, 166]
IFNγ	Pro-inflammatory cytokine	1. Its ablation restores IL33^-/-^Treg suppressive properties and IFNγ production. 2. Deletion of IFNγR renders Tregs unresponsive to this destabilizing IFNγ and fails PD-1 checkpoint blockade immunotherapy.		[37, 74]
DCs	Antigen-presenting cells	1. Inflammatory DCs are required for Treg reprogramming, driving an acquisition of pro-inflammatory phenotype. 2. Tolerogenic tumor-associated DCs resist the inflammation-induced reprogramming of Tregs in vivo.	1. Inflammatory DCs trigger IL-6 expression and cognate interaction with Tregs via MHC class II 2. Tolerogenic tumor-associated DCs express IDO, PD-L1, or semaphorin-4a to resist reprogramming. IDO blocks IL-6 production through effects on NF-IL6 (CEBP) or activating GCN2 pathway in Tregs phosphorylating the ribosomal translation factor eIF2 to alter ribosomal translation of mRNA species.	[20, 163]

Disappointingly, sometimes single or multiple blocking Treg-stabilizing signals aren’t sufficient to provide adequate anti-tumor immunity, and another possible approach would be in conjunction with active immunotherapy such as ICBs, chemotherapy, or radiation to elicit tumor killing. In I/II clinical models, the effectiveness of some IDO1 inhibitors (epacadostat, BMS-986205, KHK2455, and BGB-7204) in combination with PD-1 therapies has been demonstrated to alter the immunosuppressive environment around tumors by blocking Treg recruitment and reducing the number of Tregs in TIME, and whether this affects Treg reprogramming is unknown [170]. Unfortunately, phase 3 clinical trials of epacadostat didn’t show clinical benefit compared with pembrolizumab monotherapy in patients with advanced malignant melanoma [171]. Next, specific deletion of SCAP in Tregs reduces Pdcd1 (encoding PD-1) in combination with anti-PD-1 treatment as an effective immunotherapy. Although B16 melanoma was largely ineffective against PD-1 treatment, the absence of SCAP in Tregs sensitized mice to this immunotherapy [73]. Additionally, anti-CTLA4 checkpoint blockage raises EZH2 transcription within TIME and may facilitate the inhibitory function of TI-Tregs. Therefore, in murine cancer models, anti-CTLA4 and EZH2 inhibition could cooperate to provide strong anti-cancer immune responses [42]. Combination therapy based on synergistic mechanisms could overcome resistance to ICBs during immunotherapy. Furthermore, when Tregs are destabilized, the endogenous antigen cross-presentation could become strengthened thus promoting immune response to antigens produced by radiation and chemotherapy.

### Solution of anti-PD-1 antibody resistance by Treg reprogramming

The anti-PD-1/PD-L1 antibodies offer the dawn of cancer treatment, but the issue of resistance has been a major concern, especially in the case of solid tumors where the effectiveness is limited. Studies have found that resistance is associated with genetic mutations, increased expression of other immune checkpoints such as TIM3 and CD38, lack of tumor antigens, and impaired function of effector T cells [107]. It appears that Tregs also exert an influence on the resistance to anti-PD-1 antibodies. Wen et al. validated in the accumulation of Tregs in TIME of mice resistant to anti-PD-1 antibody was significantly higher than that of mice sensitive to anti-PD-1 antibody [172]. Likewise, in a mouse model of head and neck squamous cell carcinoma, after the application of anti-PD-L1 antibody treatment, analysis of recurrent tumors revealed a marked increase in the proportion of Tregs. To further determine whether Tregs are involved in resistance to PD-1 treatment, researchers targeted deletion Tregs with anti-CD25 antibodies, which restored anti-PD-L1 antibody-mediated anti-tumor immunity and contributed to the rejection of established tumors [173]. In addition to counteracting the tumor-killing effect, an increase in immune suppression due to the lack of PD-1 signal transduction in Tregs may also lead to tumor progression similar to hyper-progressive disease [108], a severe cancer condition characterized by rapid tumor growth after immunotherapy [174–176]. Compared to chemotherapy (5.1%), the incidence of hyper-progressive disease in non-small cell lung cancer patients receiving anti-PD-1 treatment is higher (13.8%) [109]. Detailedly, Treg-induced resistance to ICBs includes upregulation of substitute checkpoint molecules like LAG-3 and TIM-3 in Tregs [177], and increased adenosine with strong immunosuppressive effects, derived from Tregs apoptotic during PD-1 therapy.

Tregs play a crucial role in the resistance to anti-PD-1/PD-L1 antibodies. Depleting or reprogramming Tregs may improve the effectiveness of anti-PD-1/PD-L1 antibodies, offering hope for alleviating the challenge of resistance. It has been demonstrated that most TI-Tregs lose their inhibitory function and produce IFNγ after disruption of the CBM complex. This enhances the efficacy of PD-1 blockade that would otherwise not respond to anti-PD-1 monotherapy [24]. Jacquelot and colleagues confirmed that pharmacological or genetic disruption of nitric oxide synthase 2 sustained PD-1 blockade for long-term control of tumors, through decreasing Treg and DC activation [178]. Including Treg depleted (anti-CTLA-4) or destabilized (EZH2 inhibitor), restores systemic immune state and PD-1 treatment responsiveness [179].

### Future directions for TI-Tregs to improve research efficiency

The recent accelerated progress in nanodrug technology has yielded unique insights into the safety and long-term viability of cancer treatment. Many nanomedicines have been used to reduce Treg numbers and address Tregs, such as nanoscale formulations of paclitaxel and IL-2-conjugated nanoparticles. This means that we could leverage nanomedicines for precise delivery, enhanced cellular uptake, and other related benefits. Prospects for tailoring nanocarriers to administer Treg reprogramming agents hold promise for alleviating systemic toxicity and elevating drug concentrations within TIME [180]. Concurrently, it is imperative to exercise caution regarding the impact on other constituents within TIME.

Recently, state-of-the-art engineered 3D ex vivo models have the capability to replicate the intricate structures and functional characteristics of TIME, a dynamic ecological system. In contrast to conventional 2D cell culture assays and in vivo animal models utilized in cancer research, this platform presents no ethical concerns and effectively bridges the gap between oversimplified 2D systems and animal models. Notably, the composition of the TIME in animal models significantly differs from that in humans, and these models could more faithfully represent human tumor behavior. Consequently, they enhance our comprehension of cancer biology and facilitate the development of more efficacious treatment strategies, resulting in more precise and effective treatment outcomes. In recent years, the advent of engineered 3D ex vivo tissue models has markedly advanced the accurate modeling of human diseases, particularly cancer. Thus, tumor/organ-on-a-chip platforms, as more precise and realistic models, hold significant promise for investigating immune suppression mechanisms in individual TIME and Treg reprogramming therapies [181].

## Conclusion

Tregs suppress immune responses and promote tumor development. Although countless researchers are conducting clinical studies on Tregs, the focus is on Treg depletion, and there is little research on Treg reprogramming and its link to tumor treatment lists the representative portions (Table 3).

**Table 3:** Representative Treg clinical trials for tumors

NCT Number	Phases	Study Status	Conditions	Interventions	Target name	Study Results
NCT04158583	PHASE1	TERMINATED	Solid Tumors	DRUG: RO7296682	CD25	YES
NCT01929486	PHASE1	UNKNOWN	Solid Tumor	BIOLOGICAL: Mogamulizumab	CCR4	NO
NCT05537740	PHASE1	RECRUITING	Advanced Solid Tumors	DRUG: BAY3375968|DRUG: Pembrolizumab	CCR8	NO
NCT01155505	PHASE1	UNKNOWN	Advanced Solid Tumors	DRUG: Lenalidomide (CC-5013)	--	NO
NCT02705703	PHASE1|PHASE2	WITHDRAWN	Cancer|Metastatic Solid Malignancies	BIOLOGICAL: TAPA-pulsed DC vaccine	DCs	NO
NCT03601611	NA	COMPLETED	Solid Tumor|Colitis|Arthritis	DRUG: Tocilizumab (RoACTEMRA)	IL-6	NO
NCT02977156	PHASE1	COMPLETED	Metastatic Tumor|Advanced Tumor	BIOLOGICAL: Pexa-Vec|DRUG: Ipilimumab	CTLA4	NO
NCT02946671	PHASE1	COMPLETED	Gastric Cancer|Esophageal Cancer|Lung Cancer|Renal Cancer|Oral Cancer	BIOLOGICAL: Mogamulizumab|BIOLOGICAL: Nivolumab	CCR4, PD-1	NO
NCT02453620	PHASE1	ACTIVE_NOT_RECRUITING	Anatomic Stage III Breast Cancer AJCC v8|Anatomic Stage IV Breast Cancer AJCC v8|Breast Adenocarcinoma|Invasive Breast Carcinoma|Malignant Solid Neoplasm	PROCEDURE: Biopsy|OTHER: Blood Sample|PROCEDURE: Bone Scan|PROCEDURE: Computed Tomography|DRUG: Entinostat|BIOLOGICAL: Ipilimumab|BIOLOGICAL: Nivolumab|OTHER: Pharmacogenomic Study|OTHER: Pharmacological Study|PROCEDURE: Positron Emission Tomography	CCR4, PD-1	NO
NCT05200559	PHASE1|PHASE2	RECRUITING	Epithelial Ovarian Cancer	DRUG: Pembrolizumab|DRUG: E7777	PD-1, CD25	NO
NCT05469490	PHASE1	WITHDRAWN	Advanced Solid Tumors	RADIATION: Stereotactic Body Radiotherapy (SBRT)|DRUG: navoximod|DRUG: NLG802 (indoximod Prodrug)	--	NO
NCT03421353	PHASE1	ACTIVE_NOT_RECRUITING	Advanced Solid Tumours	DRUG: AZD9150|DRUG: Durvalumab|DRUG: Cisplatin|DRUG: 5-Flourouracil|DRUG: Carboplatin|DRUG: Gemcitabine|DRUG: Nab-paclitaxel	PD-1, STAT3	NO
NCT05537740	PHASE1	RECRUITING	Advanced Solid Tumors	DRUG: BAY3375968|DRUG: Pembrolizumab	CCR8	NO
NCT00128622	PHASE1	COMPLETED	Breast Cancer|Colorectal Cancer|Lung Cancer|Pancreatic Cancer|Unspecified Adult Solid Tumor, Protocol Specific	BIOLOGICAL: denileukin diftitox|BIOLOGICAL: recombinant fowlpox-CEA(6D)/TRICOM vaccine|BIOLOGICAL: therapeutic autologous dendritic cells	CD25	NO
NCT02404441	PHASE1|PHASE2	COMPLETED	Melanoma|Non-small Sell Lung Cancer (NSCLC)|Triple Negative Breast Cancer|Anaplastic Thyroid Cancer|Other Solid Tumors	BIOLOGICAL: PDR001	PD-1	YES

It is effective to reprogram Tregs for the treatment of cancer, which not only impairs Treg suppression but also converts them into pro-inflammatory phenotypes constituting a novel source of anti-tumor effector activity. Tumors with low mutation load and neoantigen levels, such as CBM complex, aren’t easily recognized thus limiting immune activation. In contrast, the TCR pool of Tregs is favored for the recognition of self-antigens and doesn’t require neoantigenic epitopes to recognize tumor cells. Therefore, pro-inflammatory Tregs acquired after reprogramming perform a vital role in tumor-killing immune activation [37]. Moreover, many of the molecules described here aren’t exclusive to TI-Tregs, and the high similarity between Tregs and other lymphocytes makes the selectively targeted suppression of intratumor Tregs difficult. Targeting these pathways in other lymphocytes may occasionally benefit cancer immunotherapy, but it may also occasionally impede the activity of beneficial immune cells leading to antagonizing the anti-tumor response. When Treg reprogramming occurs in the periphery, systemic autoimmunity is triggered by interfering with the role of Tregs in maintaining peripheral homeostasis. Consequently, current and future challenges include determining which changes in factors selectively induce specific subpopulations of Tregs in TIME but have limited effects on peripheral Tregs, as well as similarly enhancing cancer immune responses when these changes occur in other cell types. Furthermore, the preclinical research in animal models or in vitro cellular served as the main foundation for this review. Although there are signs of undergoing Treg reprogramming in humans, it remains to be established whether this also applies to human tumors treated with immunotherapy. Tumor immunotherapy against Tregs remains both challenging and promising. To minimize the potential for severe adverse reactions and increase the relevance of research findings to human tumors, it is advisable to utilize advanced technologies such as nanoparticle delivery systems and innovative tumor/organ-on-a-chip platforms in the investigation of Treg reprogramming.

## Data Availability

No datasets were generated or analysed during the current study.

## Abbreviations

TIME: tumor immune microenvironment
Tregs: regulatory T cells
ICBs: immune checkpoint blockades
cTreg: central regulatory T cell
eTreg: effector regulatory T cell
nTreg: natural regulatory T cell
iTreg: induced regulatory T cell
TI-Treg: tumor-infiltrating regulatory T cell
CTLA-4: cytotoxic T-lymphocyte-associated protein 4
PD-1: programmed death receptor 1
CTLs: cytotoxic T lymphocytes
IFN: interferon
Ezh2: enhancer of zeste homolog 2
Foxp3: Forkhead box protein P3
CNS: Conserved non-coding sequence
TET: ten-eleven translocation proteins
TNF: tumor necrosis factor
Tfr: Follicular regulatory T
Tfh: T follicular helper
TIGIT: T cell immunoglobulin and ITIM domain
CCR: C-C Motif Chemokine Receptor
Blimp1: B lymphocyte-induced maturation protein 1
STAT: signal transducers and activators of transduction
SREBPs: Sterol regulatory element-binding proteins
SCAP: SREBP cleavage-activation protein
PI3K: Phosphoinositide 3-Kinase
IKZF2: IKAROS Family Zinc Finger 2
Nr4a: nuclear receptor subfamily 4, group A
GITR: glucocorticoid-induced TNFR-related gene
NF-κB: nuclear factor kappa-B
mTOR: mammalian target of rapamycin
AKT: protein kinase, strain AK, Thymoma
Nrp-1: Neuropilin-1
PTEN: phosphatase and tensin homolog deleted on chromosome ten
Sema4a: semaphorin 4 A
CARD11: caspase recruitment domain 11
BCL10: B cell lymphoma/leukemia protein10
MALT1: Mucosa-­associated lymphoid tissue protein 1, also termed paracaspase 1
TRAF6: TNF receptor-associated factor 6
miRNAs/miRs: microRNAs
lncRNAs: long noncoding RNAs
Foxo1: forkhead box o1
IL: interleukin
ROR: RAR-related orphan nuclear receptor
IDO: indoleamine 2,3 dioxygenase
DCs: dendritic cells
